# Ingrowing Eyelashes

**Published:** 1883-07

**Authors:** William Oliver Moore


### II.—INGROWING EYELASHES—TRICHIASIS.

---

REPORTED BY WILLIAM OLIVER MOORE, M.D.

---

Recently I had brought to me a fine blooded dog, suffering with an inflammation of the left eye. The animal appeared to suffer considerable pain, and from the eye tears were streaming. On inspecting the eye I found the comjunctiva of the eye-ball reddened, and especially so at the outer and upper part. The cornea was also quite milky on the outer half. On more careful examination the cause for these appearances of the eye-ball was found to be the inserted eyelashes of the outer half of the upper lid. The constant rolling of these hairs had caused the irritation of the globe. The cause of the inversion was the cutting of the eyelashes by the attendant, and when they grew out again, instead of taking their proper direction, they grew in toward the eye-ball. Moral—Never cut eyelashes.

Treatment consisted in cutting out an oval piece of skin from the upper eyelid directly over the site of the inverted lashes, the piece about one-fourth of an inch wide and one-half inch long, and then bringing the cut sarfaces together by sutures. This procedure turns the lid edge outward, and the hairs thus turn in the proper direction. The dog made a good recovery, and was entirely relieved.

